# A distinctive subcortical functional connectivity pattern linking negative affect and treatment outcome in major depressive disorder

**DOI:** 10.1038/s41398-024-02838-7

**Published:** 2024-03-05

**Authors:** Yan-Kun Wu, Yun-Ai Su, Lin-Lin Zhu, ChaoGan Yan, Ji-Tao Li, Jing-Yu Lin, JingXu Chen, Lin Chen, Ke Li, Dan J. Stein, Tian-Mei Si

**Affiliations:** 1 Peking University Sixth Hospital, Peking University Institute of Mental Health, NHC Key Laboratory of Mental Health (Peking University), National Clinical Research Center for Mental Disorders (Peking University Sixth Hospital), Beijing, 100191 China; 2 CAS Key Laboratory of Behavioral Science, Institute of Psychology, Beijing, China; 3 Beijing HuiLongGuan Hospital, Peking University HuiLongGuan Clinical Medical School, Beijing, 100096 China; 4 PLA Strategic support Force Characteristic Medical Center, Beijing, 100101 China; 5 Neuroscience Institute, Department of Psychiatry and Mental Health, South African Medical Research Council (SAMRC), Unit on Risk and Resilience in Mental Disorders, University of Cape Town, Cape Town, South Africa

**Keywords:** Depression, Human behaviour, Predictive markers

## Abstract

Major depressive disorder (MDD) is associated with functional disturbances in subcortical regions. In this naturalistic prospective study (NCT03294525), we aimed to investigate relationships among subcortical functional connectivity (FC), mood symptom profiles and treatment outcome in MDD using multivariate methods. Medication-free participants with MDD (*n* = 135) underwent a functional magnetic resonance imaging scan at baseline and completed posttreatment clinical assessment after 8 weeks of antidepressant monotherapy. We used partial least squares (PLS) correlation analysis to explore the association between subcortical FC and mood symptom profiles. FC score, reflecting the weighted representation of each individual in this association, was computed. Replication analysis was undertaken in an independent sample (*n* = 74). We also investigated the relationship between FC score and treatment outcome in the main sample. A distinctive subcortical connectivity pattern was found to be associated with negative affect. In general, higher FC between the caudate, putamen and thalamus was associated with greater negative affect. This association was partly replicated in the independent sample (similarity between the two samples: *r* = 0.66 for subcortical connectivity, *r* = 0.75 for mood symptom profile). Lower FC score predicted both remission and response to treatment after 8 weeks of antidepressant monotherapy. The emphasis here on the role of dorsal striatum and thalamus consolidates prior work of subcortical connectivity in MDD. The findings provide insight into the pathogenesis of MDD, linking subcortical FC with negative affect. However, while the FC score significantly predicted treatment outcome, the low odds ratio suggests that finding predictive biomarkers for depression remains an aspiration.

## Introduction

Major depressive disorder (MDD) is the leading cause of disability worldwide [1], two-thirds of patients failing to achieve remission after initial antidepressant treatment [2]. Two major challenges are the heterogeneity of clinical symptomatology [3] and the need to develop more effective treatments [2]. To address these challenges, a better understanding of the pathophysiology of MDD is needed.

Since earlier studies in the fields of affective neuroscience have established the importance of the subcortical regions underlying emotion processing and regulation, abundant brain imaging studies have been examining how abnormalities of these regions may be implicated in the pathogenesis of MDD [4, 5]. Structural studies demonstrated hippocampal volumetric reductions as illness progression markers [6, 7]. Functional studies indicated enhanced amygdala (AMG) responses to negative stimuli [8–11] but blunted striatal responses to reward in MDD [12–14], which was thought to be linked to negative emotion processing bias and consummatory anhedonia, respectively. It seems that key subcortical forebrain regions (e.g. AMG and ventral striatum) are associated with core emotional symptoms in MDD. The thalamus, which conveys information between subcortical forebrain regions [15], has been linked to fear processing [16, 17] and is thus receiving increasing interest in anxiety and fear-related disorder [18, 19]. Gray matter abnormalities in the thalamus have also been found in a large sample of MDD and in relation to somatic symptoms [20]. Despite this progress, the symptom-related effects on subcortical regions remain obscure. The extent to which the subcortical functional architecture is implicated in a specific mood symptom profile remained ambiguous.

Neuroimaging studies have also suggested that striatal, hippocampal, thalamic and limbic connectivity may predict/modulate antidepressant treatment response [21–24]. One of our studies indicated that the striatal functional connectivity (FC) with prefrontal cortex is modulated by antidepressant treatment, whose changes partly underlie symptomatic improvement [25]. While such a biomarker-based approach has sought to customize individualized biological markers for treatment responses, recent evidence has suggested that positive and negative emotions and depressive symptoms may predict antidepressant treatment response, respectively [26, 27]. It is possible that a combination of subcortical connectivity and mood symptom profile may reach better prediction of treatment response. It is noteworthy that a recent groundbreaking work has implicated individualized subcortical FC score as a treatment response predictor in schizophrenia [28]. Using individualized FC to predict antidepressant outcomes has also been proved in MDD by a more recent work [29]. Hence, exploring the association between individualized subcortical FC and treatment outcome may provide novel insight into neural substrates of psychopathology and motivated the development of therapeutic targets. A rigorous examination of the relationship between subcortical FC and the specific mood symptom profile along with the contribution of this association to predict the treatment outcomes may shed new light on the pathophysiology and treatment of MDD.

Advances in high-resolution atlasing and segmentation have also provided new opportunities to explore the role of the subcortex in the pathophysiology and treatment of MDD. A novel subcortical atlas with comprehensive parcellation at the subregion level was developed recently [30] and has been utilized to investigate the illness- and treatment-related effects on subcortical regions in schizophrenia [31]. In this study, by using this novel subcortical atlas, we first aim to examine whether subcortical FC is associated with specific mood symptom profile in MDD. If so, which distinctive FC pattern contributes to the association? The secondary goal is to explore whether a personalized neuroimaging marker based on the symptom-related subcortical FC pattern can predict antidepressant response after an 8-week period of antidepressant treatment. We expected that the distinctive subcortical FC pattern that was associated with a specific mood symptom profile would be relevant to the treatment outcome. We hypothesized that a multivariate data-driven approach would allow the demonstration of an association between subcortical connectivity and mood symptom profile, and that employment of a replication sample would provide a robust foundation for subsequent analysis of treatment outcome prediction.

## Methods

Data for the present study were derived from the Towards Neurobiology-based Diagnosis and Treatment of Affective Disorders (TNDTAD) project (NCT03294525), which is a naturalistic prospective study designed to explore biomarkers of diagnosis and predictors of treatment outcome in patients with mood disorders [32]. Medication-free patients with MDD (received no psychotropic medications for 2 weeks except for benzodiazepines) were treated with antidepressant monotherapy (i.e., selective serotonin reuptake inhibitor, serotonin and noradrenaline reuptake inhibitor and other antidepressants) at flexible doses according to national guidelines for the Prevention and Treatment of Major Depressive Disorder in China [33] and prescribers’ clinical practice for 8 weeks. Dose titration was completed in two weeks based on treatment response and side effects. The antidepressant dose remained unchanged after dose titration until the end of the 8-week treatment. These patients completed pretreatment and posttreatment clinical assessment at baseline and after 8 weeks of antidepressant treatment, respectively.

### Participants

Medication-free patients with MDD aged 18 to 55 years were enrolled from the Outpatient Department of Beijing HuiLongGuan Hospital. Diagnoses were confirmed according to DSM-IV-TR criteria for MDD by two qualified psychiatrists using the Mini-International Neuropsychiatric Interview (M.I.N.I.) [34].

Inclusion criteria for patients with MDD were: 1) total score on the 17-item Hamilton Rating Scale for Depression (HRSD-17) [35] ≥14; 2) no psychotropic medication (except for benzodiazepines) for at least 2 weeks (4 weeks for fluoxetine) before enrollment.

Exclusion criteria for all participants included: 1) lifetime or current diagnosis of other major Axis I psychiatric disorders, including psychotic disorder, bipolar disorder, alcohol/substance dependence or abuse (screened by two qualified psychiatrists using the M.I.N.I.), Axis II personality disorder or intellectual disability; 2) severe somatic diseases (such as severe cardio-cerebral vascular diseases, respiratory diseases, liver diseases, kidney diseases, or malignant tumors); 3) current pregnancy or breastfeeding; 4) electroconvulsive therapy in the last six months.

Among the one hundred and fifty-eight patients eligible after initial screening, nineteen did not complete magnetic resonance imaging (MRI) scanning due to lack of consent, inability to complete scanning or contraindications to MRI scanning. Of the 139 included patients, one presented with hypomanic symptom during the follow-up, one had incomplete clinical assessment data, one had poor quality MRI data, and one had excessive head motion. After exclusion of these patients, 135 patients remained for analysis.

Among the 135 patients, 85 were prescribed selective serotonin reuptake inhibitor, 31 were prescribed serotonin and norepinephrine reuptake inhibitor and 19 were prescribed other antidepressants such as vortioxetine, mirtazapine, agomelatine. The flowchart for patient analysis is shown in Supplementary Figure S1. At 8-week follow-up, 84 of 135 patients completed posttreatment clinical assessment. These patients did not receive any other psychotropic medications or a second antidepressant and their medication was not changed during the 8-week antidepressant treatment. The mean fluoxetine-equivalent dose for these patients was 32.91 mg/d [36].

All procedures involving human subjects/patients were approved by the independent Ethics Committee of Peking University Sixth Hospital and Human Ethics Committee of Beijing HuiLongGuan Hospital. Written informed consent was obtained from all participants prior to data collection.

### Clinical assessment

Depressive symptom severity was assessed using the clinician-rated HRSD-17, which includes items assessing emotional, cognitive, and neurovegetative symptoms. Mood symptoms were also measured using the self-rated Positive And Negative Affect Schedule (PANAS) [37], a well-validated assessment of current mood state (i.e., past two weeks in this study). The PANAS contains 10 positive affect (PA) and 10 negative affect (NA) items. The total set of 37 items measured at baseline was constructed as a mood symptom profile, generating a 37 × 1 vector for subsequent analysis.

### MRI acquisition and processing

MRI data of the main and replication sample were acquired on a Siemens Prisma 3.0 T MRI scanner in the Beijing Huilongguan Hospital and on a Siemens 3.0 T Trio scanner in the 306th Hospital, respectively. The details of MRI data acquisition and processing are described in the Supplementary methods. To minimize the head motion effect on functional connectivity, scrubbing was used to replicate the main findings. Details are described in the Supplementary methods.

### Subcortical resting-state functional connectivity

**Fig. 1:**
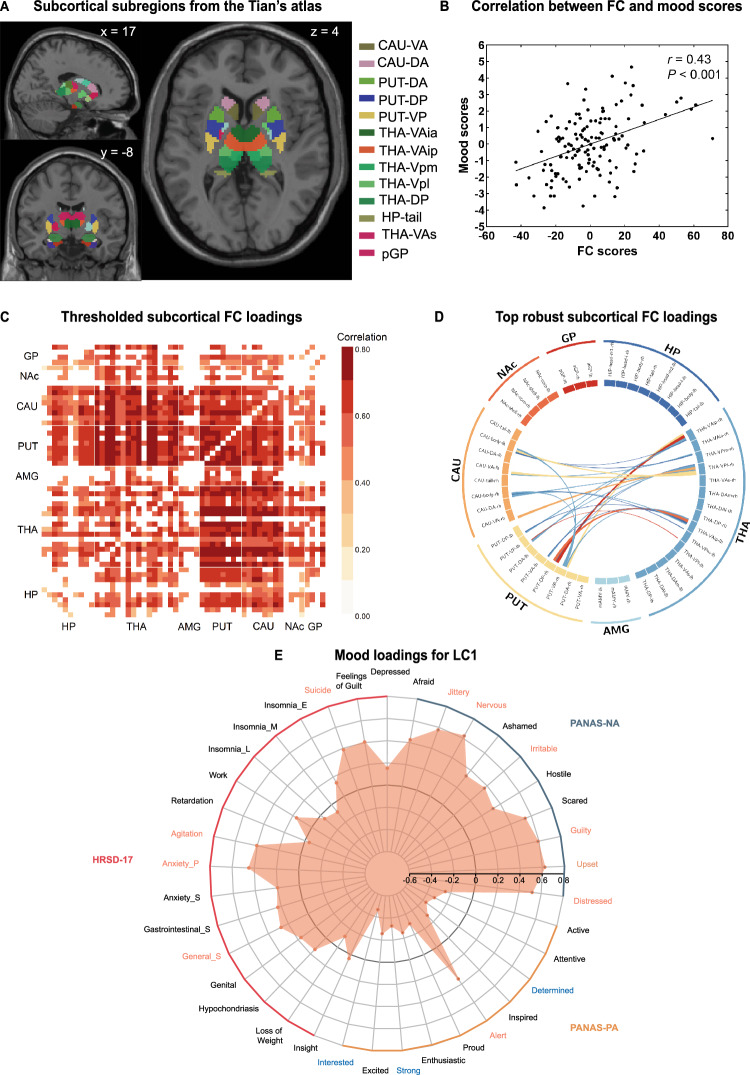
Partial least squares correlation analysis in the main sample. **A** Illustration of the subcortical subregions from the Tian’s atlas [30]. **B** Correlations between individual-specific functional connectivity (FC) scores and mood scores for the significant LC (i.e., the first LC, LC1). **C** Thresholded correlations between individual-specific original FCs and FC scores, whereby only the reliable connections (i.e., absolute value of bootstrap ratio of the FC greater than 2) that show significant FC loadings are shown. None of the thresholded correlations was negative. **D** Subcortical connections with top 5% robust FC loadings showing in the chord diagram, whereby only those connections whose absolute values of bootstrap ratio are greater than 3 and whose FC loadings are significant are shown. **E** Mood loadings for LC1. Items in PANAS-PA, PANAS-NA, and HRSD-17 are inside the orange, blue, and red arcs, respectively. Item spots inside the black circle indicates that the mood item is negatively associated with mood score and vice versa. Mood item labels with reliable contribution to the LC1 and significant mood loadings are shown in orange (or blue), indicating that higher degree of the mood item is significantly and positively (or negatively) associated with LC1 and also reliably contributed to the LC1. HP hippocampus, THA thalamus, AMG amygdala, PUT putamen, CAU caudate nucleus, NAc nucleus accumbens, GP globus pallidus, pGP posterior globus pallidus, VA ventroanterior, DA dorsoanterior, DP dorsoposterior, VP ventroposterior, THA-VAia anterior division of inferior ventroanterior thalamus, THA-VAip posterior division of inferior ventroanterior thalamus, THA-Vpm medial ventroposterior thalamus, THA-Vpl lateral ventroposterior thalamus, THA-VAs superior ventroanterior thalamus, Insomnia_E Insomnia Early, Insomnia_M Insomnia Middle, Insomnia_L Insomnia Late, Anxiety_P Psychological Anxiety, Anxiety_S Somatic Anxiety, Gastrointestinal_S Somatic Symptoms (gastrointestinal), General_S Somatic Symptoms (general).

The subcortical network comprises the hippocampus (HP), thalamus (THA), AMG, putamen (PUT), caudate nucleus (CAU), nucleus accumbens (NAc) and globus pallidus (GP) [38]. These structures were defined according to recent work by Tian et al. [30], with semiautomatic delineation using gradientography, a functional MRI analog of diffusion MRI tractography that enables the quantification of subcortical connectivity gradients. The new atlas is significantly more homogeneous than existing MRI-based parcellations of the entire subcortex [39, 40], the HP [41], the THA [42] or the striatum [43] as well as histology-based HP parcellation [44]. The atlas allows fine parcellation of 54 subregions (Fig. 1A). Blood oxygen level-dependent signals were extracted from the 54 subregions and Pearson’s correlation coefficients (i.e., subcortical FC values) were calculated between mean time series of each brain region and transformed to *z*-scores using the Fisher r-to-z formula. A 54 × 54 FC matrix was generated for subsequent analysis.

### Subcortical FC-mood symptom association analysis

Partial least squares (PLS) correlation analysis is a multivariate data-driven statistic technique that has been used in several neuroimaging domains to examine the relationship between brain activity and behavior [45]. We used PLS to assess multivariate associations between subcortical FCs (i.e., the 54 × 54 FC matrix) and the mood symptom profile (i.e., the 37 × 1 vector). The goal of PLS analysis is to extract a set of latent components (LCs) that maximally explain covariance between the two sets of variables, which are optimal linear combinations of the original variables. Details about the PLS analysis see Supplementary methods. The LCs were ranked by the amount of covariance that each contributes, with each LC comprising a FC salience and a mood salience. The saliences are akin to loadings in the principal component analysis [46]. The *P* value of each LC was determined using permutation tests (5000 times). A *P*-value threshold of 0.05 was selected according to Bussy et al. [47].

To test the reliability of each FC measure and mood item in the significant LCs, we generated 5000 bootstrap samples. Bootstrap ratio (BSR) was computed by dividing each pair of saliences by its bootstrap estimated standard deviation. The BSR is akin to a *z*-statistic, of which over a specific threshold is considered reliable for a measurement. The FC/mood BSR represents the reliability of the FC measure/mood item contributing to the corresponding LC. A BSR threshold of 2, analogous to a *P*-value of 0.05, was applied according to Krishnan A et al. [45].

For each participant, by linearly projecting the individual subcortical FCs and mood symptom profile onto the respective salience of each LC, we obtained individual FC scores and mood scores for each LC. That is, FC scores and mood scores reflect the participants’ individual subcortical FCs and mood symptom profile contribution to each LC.

To interpret the significant LCs, FC loadings were computed for each LC between the original subcortical FCs and individual-specific FC scores, and mood loadings were computed for each LC between the original mood symptom profile and individual-specific mood scores, using Pearson’s correlations according to Kebets et al. [46]. For a given LC, the loadings indicate the contribution of an FC measure or a mood item to the subcortical FC-mood symptom profile covariation. We estimated confidence intervals on the loadings to confirm the significance by a bootstrap procedure (5000 times).

PLS analysis was performed using the my-pls toolbox (https://github.com/danizoeller/myPLS) implemented in Matlab.

### Replication analysis

To validate the robustness of the subcortical FC to mood symptom profile association, an independent sample recruited from another study by our team was analyzed [48]. The same inclusion and exclusion criteria, data collection and processing pipeline were adopted, with the exception of the inclusion criterion of symptom severity. Patients with MDD were required to score at least 18 in HRSD-17. MRI data acquisition is described in the Supplementary methods. To verify the nature of the subcortical FC to mood symptom profile association, the PLS analysis was independently repeated in the replication sample.

We applied two strategies to assess the generalizability of the PLS association. First, the similarity in FC/mood loadings of the significant LCs between the two samples was measured by Pearson’s correlations. Second, we applied the FC salience and mood salience obtained in the main sample to the replication sample. That is, predicted FC and mood scores of the replication sample were obtained by linearly projecting the original FCs and mood symptom profile onto the respective salience of the main sample. The correlation between predicted and observed FC/mood scores was measured in the replication sample.

### Prediction analysis

Previous study indicated that individualized subcortical FC score could comprise an objective biomarker to predict treatment outcome [28]. Thus, we were interested in whether individual FC score, which was inherently associated with specific mood symptom profile, would be a predictive functional MRI marker of treatment outcome, which was assessed after eight-week antidepressant treatment. We thus performed general linear models (implemented in Matlab) with FC scores for significant LCs as predictors to examine the prediction effect of the subcortical FC pattern on treatment outcome in the discovery sample. Existing evidence suggests that age of onset [49] and duration of the current episode [49] may predict treatment outcome, thus we included these variables as predictors. Treatment outcome was measured in two ways: a) percentage reduction in HRSD-17 score, i.e., ΔHRSD-17 = (baseline HRSD-17 - posttreatment HRSD-17) / baseline HRSD-17 × 100% [50], and b) posttreatment HRSD-17 score. Stepwise fitting was used to determine the only relevant variables that fit the models. To examine whether clinically meaningful outcomes were predicted by these variables, logistic regression was conducted using stepwise fitting. In accordance with clinical practice, treatment outcomes were defined in two ways: a) treatment response, >50% reduction in posttreatment HRSD-17 score, and b) depression remission, posttreatment HRSD-17 score ≤7. Standardized regression coefficients B are reported for the linear regressions and Odd’s Ratios (OR) for the logistic regressions. To examine whether the prediction effect could be affected by head motion, we repeated the above models adding the head motion as a predictor variable.

## Results

Table 1 lists separately the demographic and clinical characteristics of subjects in the TNDTAD study. All participants are Asian. Most patients with MDD were first episode MDD (57.0%), and mean HRSD-17 total score was 21.3, indicating moderate to severe depression.

**Table 1:** Demographics, clinical characteristics and clinical measurements of subjects.

	TNDTAD study (*n* = 135)		Replication sample (*n* = 74)		*P* value
	Mean	SD	Mean	SD	
Age	29.4	8.4	31.6	9.3	0.072
BMI	22.0	3.3	22.0	3.1	0.893
Edu (years)	15.8	2.2	15.1	3.4	0.110
Age of onset^a^	27.7	8.8	/	/	/
Total disease duration (months)^a^^,^^b^	21.9	34.4	/	/	/
Duration of current episode (months)^a^	4.6	4.4	9.5	16.5	0.032
Total score of childhood trauma	41.5	11.0	42.0	11.3	0.770
HRSD-17 total score	21.3	4.3	22.8	4.3	0.018
PANAS-PA score	18.1	4.8	19.8	5.1	0.016
PANAS-NA score	27.5	6.8	29.9	6.6	0.015
fluoxetine-equivalent dose (mg/day)	32.9	10.1	/	/	/

	N	%	N	%	
Female	93	68.9	47	63.5	0.429
First Episode MDD	77	57.0	64	86.5	<0.001
Psychosis symptoms^a^	3	2.3	2	2.7	1.000
Family history of psychiatric disorders^b^	17	12.6	7	9.5	0.497
SSRI	85	63.0	/	/	/
SNRI	31	23.0	/	/	/
Other antidepressants	19	14.0	/	/	/

^a^Due to missing information, the number of invalid data in TNDTAD study is as follows: Melancholic MDD (*n* = 12), Psychosis symptoms (*n* = 6), Age of onset (*n* = 1), Total disease duration (*n* = 1), Duration of the current episode (*n* = 32); The number of invalid data in replication sample is as follows: Duration of current episode (*n* = 16). Percentage was calculated based on valid data.

^b^Total disease duration was defined as summed duration of one or several depressed episodes by the end of recruitment; Family history of psychiatric disorders was defined as having first-degree relatives that has been diagnosed as any DSM-IV-TR Axis I disorder. *HRSD-17* 17-item Hamilton Rating Scale for Depression, *PANAS* Positive and negative affect schedule, *PA* positive affect, *NA* negative score, *SSRI* selective serotonin reuptake inhibitor, *SNRI* serotonin and noradrenaline reuptake inhibitor.

### Identification of covariance patterns

#### Associated dorsal striatal and thalamic network and negative affect

Among the 37 LCs extracted by PLS analysis, only the first LC (LC1) was significant (permutation test, *P* < 0.001). LC1 accounted for 47.5% of the FC-mood covariance (Figure S2A). A significant association was found between FC and mood scores for LC1 (*r* = 0.43, *P* = 2.35 × 10^−^^6^, Fig. 1B).

FC loadings for LC1 were shown in Fig. 1C (Thresholded FC loadings) and Fig. 1D (Top robust FC loadings). Greater FC score was associated with increased subcortical FC showing a distributed pattern, but with involvement of dorsal striatum (i.e., putamen and caudate) and thalamus.

Mood loadings for LC1 are shown in Fig. 1E. Greater mood score was associated with increased anxiety (e.g., nervous, psychological anxiety, general somatic symptoms, irritable, agitation) and general and extreme negative affect (e.g., suicide, guilty, upset and distressed).

The covariance pattern was replicable after scrubbing (Supplementary results, Fig. S3).

#### Consideration of other potential contributing factors

To clarify whether the FC score and mood score were driven by demographic or clinical characteristics, we conducted correlation and group comparison analyses. No significant correlations were found between mood scores/FC scores and demographic variables (i.e., age, years of education years, BMI, and depression characteristics (i.e., total disease duration, duration of current episode, and age of onset) (Table S1). First-episode patients and recurrent patients with MDD showed no significant differences in mood score (*P* = 0.821) and FC score (*P* = 0.160). Patients with a family history of psychiatric disorders and patients without a family history of psychiatric disorders showed no significant difference in mood score (*P* = 0.816) and FC score (*P* = 0.834).

The results remained consistent after controlling potential confounds (i.e. age, sex, years of education, BMI and head motion, see Supplementary results).

#### Replication sample results

The demographic and clinical characteristics of subjects in the replication sample are listed in Table 1. Participants are Asian. No significant differences on demographic measures were observed. With the more stringent inclusion criterion of symptom severity in the replication sample, subjects in the replication sample showed higher scores on the HRSD-17, PANAS-PA, PANAS-NA. Subjects in the replication sample had longer mean duration of the current episode and higher percentage of first-episode MDD compared to subjects in the TNATAD study.

**Fig. 2:**
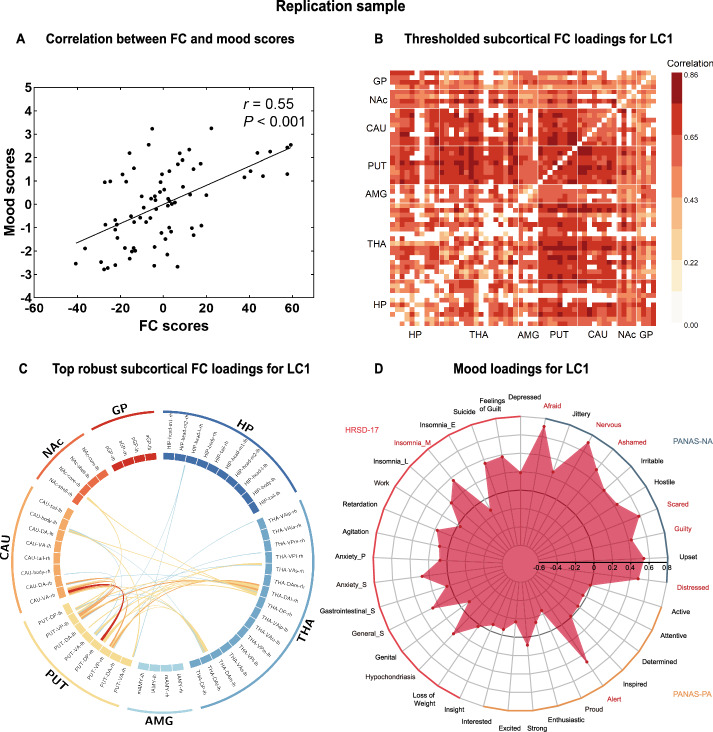
Partial least squares correlation analysis in the replication sample. **A** Correlations between individual-specific functional connectivity (FC) scores and mood scores for the significant LC (i.e., the first LC, LC1). **B** Thresholded correlations between individual-specific original FCs and FC scores, whereby only the reliable connections (i.e., absolute value of bootstrap ratio of the FC greater than 2) that show significant FC loadings are shown. None of the thresholded correlations was negative. **C** Subcortical connections with top 5% robust FC loadings showing in the chord diagram, whereby only those connections whose absolute values of bootstrap ratio are greater than 3 and whose FC loadings are significant are shown. **D** Mood loadings for LC1. Items in PANAS-PA, PANAS-NA, and HRSD-17 are inside the orange, blue, and red arcs, respectively. Item spots inside the black circle indicates that the mood item is negatively associated with mood score and vice versa. Mood item labels with reliable contribution to the LC1 and significant mood loadings are shown in red, indicating that higher degree of the mood item is significantly and positively associated with LC1 and also reliably contributed to the LC1.

Only the first LC (LC1) survived the permutation test, accounting for 47.0% of the FC-mood covariance (Figure S2B), with significant association (*r* = 0.55, *P* = 3.21 × 10^−7^, Fig. 2A) between FC and mood scores for the replication sample. The subcortical FC pattern that reliably contributed to the LC1 was similar to that seen in the TNDTAD study but was more extensive (Fig. 2B). Nonetheless, subcortical FCs that reliably contributed to LC1 and highly correlated with LC1 were connectivity within dorsal striatum, and between dorsal striatum and thalamus (Fig. 2C). The mood loading profile of LC1 (Fig. 2D) was dominated by negative affect (e.g., afraid, nervous, scared and distressed).

The loadings of the replication sample were moderately consistent with that of the main sample (*r* = 0.66, *P* = 3.39 × 10^−183^ for FC loadings, *r* = 0.75, *P* = 9.56 × 10^−8^ for mood loadings). The PLS model achieved good performance in the replication sample with moderate correlations between predicted and observed scores (*r* = 0.995, *P* < 0.001 for FC scores and *r* = 0.696, *P* < 0.001 for mood scores, Figure S4).

### Prediction effects of FC pattern on treatment outcome

Among the 84 patients, 41 patients (accounting for 48.81%) remitted and others did not. Comparisons of clinical characteristics between non-remitted and remitted patients are depicted in Table S2. Remitted patients showed shorter total disease duration and lower total score of childhood trauma.

**Fig. 3:**
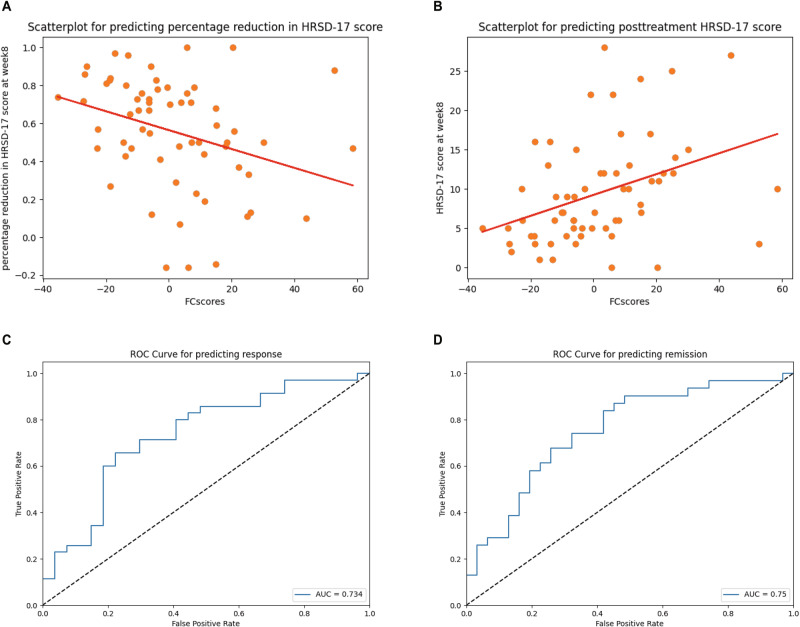
Treatment prediction analysis. **A** Scatterplot for predicting percentage reduction in HRSD-17 score at week 8. **B** Scatterplot for predicting HRSD-17 score at week 8. **C** ROC curve for predicting response to 8-week treatment. **D** ROC curve for predicting remission to 8-week treatment.

For dimensional treatment outcome prediction (Table S3), only the FC score survived stepwise fitting (B = −0.005, 95% CI = −0.009−0.001, *P* = 0.009 for predicting percentage reduction in HRSD-17 score, Fig. 3A; B = 0.133, 95% CI = 0.049–0.217, *P* = 0.002 for predicting posttreatment HRSD-17 score, Fig. 3B). In logistic regression, only the FC score survived. Although increased FC score was associated with reduced probability of responding to antidepressants, the effect size was small (odds ratio = 0.954, 95% CI = 0.922–0.987, *P* = 0.006) and with modest classification accuracy (accuracy = 0.710, AUC = 0.734, Fig. 3C). The FC score also significantly predicted remission status with relatively small effect size (OR = 0.948, 95% CI = 0.915–0.983, *P* = 0.004) and with modest classification accuracy (accuracy = 0.710, AUC = 0.754, Fig. 3D). The results remained the same after adding the head motion as a predictor variable.

## Discussion

The main finding of this work was that a distinctive aberrant subcortical functional connectivity pattern, critically involving the dorsal striatum and thalamus, is associated with increased negative affect and worse treatment outcome in MDD. The link demonstrated here between subcortical functional connectivity and negative affect is consistent with prior literature on the neurobiology of depression. However, the multivariate approach employed here, together with a replication analysis, consolidates and extends this literature.

The specific affective pathology uncovered by our analysis may be related to negative affect, including general and extreme negative affect (e.g., guilt, suicide, upset and distress) and anxiety (e.g., nervous, irritable, scared, agitation and psychological anxiety), with moderate correlation. Critically, the caudate, mainly the body part, thalamus, mainly the ventral part (ventroposterior thalamus and posterior division of ventroanterior thalamus) and the putamen was highly correlated with the specific affective pathology. One study examining positive and negative affect in healthy volunteers lends support to the involvement of putamen in negative affect [51]. Recent studies have demonstrated the multimodal abnormalities of structure and function in putamen may not only present in patients with MDD but also present in subjects in familial risk for depression, indicating putamen as a potential biomarker for depressive illness [52, 53]. Specially, the posterior putamen is specialized in habitual or automatic responding [48]. Another study mapping cognition and affect onto neurobiological substrates suggests the ventral caudate is implicated in affective functions such as pain processing [54]. Our analysis extends our knowledge of the role of the dorsal striatum in pathological negative affect. Of note, recent rodent studies have revealed that stress may trigger changes of the structure and functional connectivity of thalamus [55] and the thalamus is implicated in the contextual processing of stress-related disorder [56]. Abundant of evidence emphasizes that the thalamus plays a key role in fear-related learning [18], especially subjective fear [16]. It is possible that these subdivisions of dorsal striatum together with thalamus play an important role in the automatic processing of potential or sustained threat [57, 58] and functional abnormalities may contribute to anxiety and extends to general negative affect in MDD. Such an altered connectivity pattern was also found in treatment-seeking youth, associated with anxiety, internalizing symptoms and suicidal thoughts [58, 59]. Taken together, this evidence lends support to the coordinated activity underlying habitual and endogenous processing of negative thoughts in the absence of task demands in MDD.

The subcortical FC pattern captured by LC1 here also suggested mild to moderate correlation with other distributed subcortical regions such as NAc and HP. The symptom profile of LC1 was mildly negatively correlated with positive affect and less correlated with sleep disturbance and psychomotor retardation. These findings may be interpreted as the integrated nature of brain connectivity. Specifically, it appears that depressive symptoms may involve subcortical regions that extend across multiple neural systems [46, 57–59]. Previous evidence has suggested that the local functional activity of amygdala, ventral striatum, hippocampus and subgenual anterior cingulate drives general distress in MDD [60] and the subjective experience of fear involves distributed brain systems [16]. Nonetheless, our findings highlighted the central role of the thalamus and dorsal striatum in negative affect. Interestingly, “alert”, which is classified into positive affect, was positively correlated with LC1, indicating that it was interpreted as a negative affect in patients with MDD. Cultural diversity may partly account for the discrepancy because this item remains controversial in the Chinese version of the PANAS [61, 62], showing moderate correlation with both positive and negative affect. On the other hand, this interesting result may reflect a negative bias effect during emotion processing in MDD [63]. Consistently, previous evidence has suggested that increased level of alert is associated with anxiety [64, 65].

Though similar FC pattern and mood symptom profile were found in the replication sample, there are discrepancies. The mood symptom profile of LC1 in the replication sample was less typical, showing higher positive loading on middle insomnia, lower positive loadings on gastrointestinal and genital symptom and lower negative loadings on positive affect. The neural substrates associated with the mood symptom profile involved a wider range of regions, such as NAc and GP, indicating a diverse spectrum of symptoms that the LC1 explained [57]. Basically, certain differences in clinical characteristics exist between the two sample, for example, patients in the replication sample showed more severe depressed symptoms and higher positive and negative affect. Less emotional blunting in the replication sample might contribute to the nontypical mood profile and extensive subcortical FC pattern.

Lower individual subcortical FC score might be associated with better pharmacotherapy outcome with greater symptom improvement. Currently, clinician prescription is based primarily on subjective clinical judgement. Thus, an individualized composite score is not only a considerable objective indicator but also applicable and generalizable in clinical practice. Our findings suggested that the FC score could be interpreted as a functional MRI marker for overall mono-pharmacotherapy effects. For those who show higher composite FC score, symptoms involving negative valence might be presented [66] and more intensive follow-up and combined therapy may be important. The individual FC score reflected individualized projection of the identified subcortical FC pattern, which was associated with specific negative affect, and thus shed light on the possibility of subcortical function as predictive biomarkers, suggested by previous evidence [22–24]. Of note, the thalamus is a key deep brain stimulation targets in the treatment of neurological and psychiatric disorders [67]. One randomized controlled trial has suggested acupuncture may achieve treatment effects by modulating the functional connectivity of putamen and caudate [68]. Our findings suggested that future neuromodulation development might benefit from considering thalamus and dorsal striatum as potential treatment targets. In terms of the covariation between mood score and FC score, the distinctive subcortical FC pattern may comprise a neurobiological marker of distinct symptom profile severity as well as a neurobiological marker of response to antidepressant treatment. Nevertheless, finding predictive biomarkers remains aspirational for the field despite the small effect size.

Several limitations should be acknowledged. First, the lack of patients with diagnoses other than MDD and the lack of healthy controls restricts our findings in exploring neural substrates dimensionally associated with clinical psychopathology. Second, the open-label nature of the study with the absence of placebo make it difficult to disentangle the placebo effect or to dissect drug-specific from non-specific treatment effects [69]. Third, further validation is needed to test the robustness and effectiveness of the subcortical FC score as a predictive biomarker in a large sample. Despite these limitations, the multivariable data-driven approach employed here enabled us to detect a significant subcortical pattern related to a specific symptom profile and the employment of a replication sample allowed us to assess the robustness of our findings. Finally, there are parallel anatomical and functional cortical-subcortical circuits linking the cortex to subcortical structure. The interplay between these regions is of utmost importance, and our current study serves as a preliminary investigation, hinting at the key subcortical regions in relation to mood symptom profiles.

In summary, the emphasis here on the role of dorsal striatum and thalamus consolidates prior work on the role of these structures in altered subcortical functional connectivity in MDD. The findings here are robust to shed light on the pathogenesis of MDD, linking subcortical functional connectivity with negative affect. However, while the connectivity pattern significantly predicted treatment outcome, the low odds ratio suggests that finding predictive biomarkers for depression remains aspirational.

### Supplementary information

supplementary information

## Data Availability

The study team are available to collaborate with other research teams on receipt of a reasonable written request to access study data.
