# Supplementary material for: A distinctive subcortical functional connectivity pattern linking negative affect and treatment outcome in major depressive disorder
Source: Transl Psychiatry. 2024 Mar 5;14:136. doi: 10.1038/s41398-024-02838-7 (PMC10915152; doi:10.1038/s41398-024-02838-7)
Supplement: Supplementary file 1 — supplementary information [file 41398_2024_2838_MOESM1_ESM.docx]

**Supplementary information**

**A distinctive subcortical functional connectivity pattern linking negative affect and treatment outcome in major depressive disorder**

Wu Yan-Kun^1#^, M.B.; Su Yun-Ai^1#^, M.D.; Zhu Lin-Lin^1^, Ph.D.; Yan ChaoGan^2^, Ph.D.; Li Ji-Tao^1^, M.D.; Lin Jing-Yu^1^, M.B.; Chen JingXu^3^, M.D.; Chen Lin^3^, M.D.; Li Ke^4^, M.D.; Dan J Stein^5^, M.D.; Si Tian-Mei^1^, M.D.

^1^ Peking University Sixth Hospital, Peking University Institute of Mental Health, NHC Key Laboratory of Mental Health (Peking University), National Clinical Research Center for Mental Disorders (Peking University Sixth Hospital), Beijing 100191, China

^2^ CAS Key Laboratory of Behavioral Science, Institute of Psychology, Beijing, China

^3^ Beijing HuiLongGuan Hospital, Peking University HuiLongGuan Clinical Medical School, Beijing 100096, China

^4^ PLA Strategic support Force Characteristic Medical Center, Beijing 100101, China

^5^ Neuroscience Institute, Department of Psychiatry and Mental Health, South African Medical Research Council (SAMRC), Unit on Risk and Resilience in Mental Disorders, University of Cape Town, Cape Town, South Africa

^#^ Wu YK and Su YA contributed equally to this work.

Corresponding Authors: Si Tian-Mei (e-mail: si.tian-mei@163.com; address: No. 51 Hua Yuan Bei Road, Haidian District, Beijing 100191, China) and Su Yun-Ai (e-mail: suyunai@163.com; address: No. 51 Hua Yuan Bei Road, Haidian District, Beijing 100191, China)

**Content summary**

[Supplementary methods 3](#_Toc151069710)

[**MRI data acquisition** 3](#_Toc151069711)

[**MRI data processing** 4](#_Toc151069712)

[**Partial least squares (PLS) analysis** 4](#_Toc151069713)

[Supplementary results 6](#_Toc151069714)

[**Identification of covariance patterns after scrubbing** 6](#_Toc151069715)

[**Consideration of Other Potential Contributing Factors** 6](#_Toc151069716)

[Supplementary figures 7](#_Toc151069717)

[**Figure S1. Flow chart depicting the patient analysis procedure.** 7](#_Toc151069718)

[**Figure S2. Covariance explained by each latent component obtained with the PLS analysis for the TNATAD study (A) and replication sample (B).** 8](#_Toc151069719)

[**Figure S3. FC and mood loadings in the main sample after scrubbing.** 9](#_Toc151069720)

[**Figure S4. Correlations between predicted and observed FC/mood scores in the replication sample.** 11](#_Toc151069721)

[Supplementary tables 12](#_Toc151069722)

[References 15](#_Toc151069723)

# Supplementary methods

**MRI data acquisition**

Subjects in the mian sample were scanned on a Siemens Prisma 3.0T magnetic resonance imaging (MRI) scanner in the Beijing Huilongguan Hospital. The functional images lasted seven minutes using the T2*-weighted gradient-echo echo-planar imaging (EPI) sequence with parameters: repetition time (TR) = 2000 ms; echo time (TE) = 30 ms; in-plane resolution, 3.8 × 3.8 mm^2^; matrix, 64 × 64; field of view (FOV), 240 × 240 mm^2^; flip angle, 70°; 32 slices; 180 volumes; thickness, 5.0 mm; head coil: 64 ch. The structural images were obtained using T1-weighted magnetization-prepared rapidly acquired gradient-echo (MPRAGE) sequence with parameters: TR = 2000 ms; echo time (TE) = 2.28 ms; in-plane resolution, 1.0 × 1.0 mm^2^; matrix, 256 × 256; field of view (FOV), 256 × 256 mm^2^; flip angle, 9°; 192 slices; thickness, 1.0 mm; head coil: 64 ch. During the resting-state scanning, participants were instructed to relax with their eyes closed, avoid head motion and not to fall asleep. A simple questionnaire was performed to confirm that the participants had followed the instructions. Only eligible participants were included.

*Subjects* *in the replication analysis*

Subjects were scanned on a Siemens 3.0T Trio scanner in the 306th Hospital. The functional images lasted seven minutes using the T2*-weighted gradient-echo echo-planar imaging (EPI) sequence with parameters: repetition time (TR) = 2000 ms; echo time (TE) = 30 ms; in-plane resolution, 3.3 × 3.3 mm^2^; matrix, 64 × 64; field of view (FOV), 210 × 210 mm^2^; flip angle, 90°; 30 slices; 210 volumes; thickness/gap, 4.0 mm/0.8 mm; head coil: 32 ch. The structural images were obtained using T1-weighted magnetization-prepared rapidly acquired gradient-echo (MPRAGE) sequence with parameters: TR = 2300 ms; echo time (TE) = 3.01 ms; in-plane resolution, 1.0 × 1.0 mm^2^; matrix, 256 × 256; field of view (FOV), 240 × 256 mm^2^; flip angle, 9°; 176 slices; thickness, 1.0 mm; head coil: 32 ch.

**MRI data processing**

The MRI data were preprocessed with DPABISurf toolbox (DPABISurf_V1.2_190919, http://rfmri.org/DPABISurf)(1) based on Statistical Parametric Mapping (SPM12, <http://www.fil.ion.ucl.ac.uk/spm>). After removing the first 10 volumes, the remaining 200 volumes were corrected for different slice acquisition times and head motion with six-parameter rigid-body transformation. Nuisance covariates were regressed out from the time series, including the Friston-24 parameters of head motion, time series extracted from cerebrospinal fluid regressors and white matter regions and linear trends. The structural images were segmented using fast (FSL 5.0.9) (2) and normalized to Montreal Neurological Institute (MNI) space using antsRegistration (ANTs 2.2.0). The derived functional images were coregistered to the corresponding structural images and normalized to MNI space with the warping parameters, and resampled to 3 mm cubic voxels. Then the images were band-pass filtered (0.01 - 0.1Hz) and spatially smoothed with full width at half maximum of 6 mm. To minimize head motion effects on the data, a rigorous threshold for mean frame-wise displacement (FD, measured by Jenkinson method (3)), no larger than 0.3 mm, was applied. One MDD patient was excluded (Supplementary Figure S1). No MDD patient was excluded in the replication sample.

Though we regressed out the 24 Friston head motion parameters in nuisance regression, considering the crucial impact of motions on data quality, we processed the fMRI data with scrubbing method. Two scrubbing strategies can be employed, i.e., volume removing and spike regression. In view of concern that volume removing may result in different numbers of volumes across subjects, thereby impacting the accuracy of functional connectivity, we used the overthreshold time points as a separate regressor (4). The overthreshold time points were defined as over 0.3 mm by the FD_Jenkinson method (3) as mentioned above.

**Partial least squares (PLS) analysis**

Partial least squares (PLS) analysis is a multivariate data-driven statistical technique that aims to extract a set of latent components (LCs) that maximally explain covariance between two sets of variables (subcortical FCs and mood symptom profile in this case). We used the my-pls toolbox (https://github.com/danizoeller/myPLS) implemented in Matlab according to Kebets et al. (5) and Krishnan A et al. (6). The lower triangle of the subcortical functional connectivity (FC) matrix (i.e. 1431 FC values) was considered in subsequent analyses. The PLS analysis in the TNDTAD study was calculated as follows. The subcortical FCs and mood symptom profile are stored in matrices *X* (matrix size 135 × 1431) and *Y* (matrix size 135 × 37), respectively. The PLS seeks to maximizes the covariance between matrices *X* and *Y* by extracting latent components (LCs). After z-scoring *X* and *Y*, we computed the covariance matrix *R*:

*R* = *Y^T^* × *X*

Singular value decomposition of the correlation matrix *R* was computed as:

𝑅 = 𝑈 × 𝑆 × 𝑉*^T^*

U and V are mood salience and subcortical FC salience (i.e. the singular vectors, akin to loadings in principal component analysis), while S is a diagonal matrix containing the singular values. The LCs were ranked by the amount of covariance that each contributes. Each LC is characterized by a distinct subcortical FC pattern (i.e., subcortical FC salience) and a distinct mood symptom profile (i.e., mood salience). The significance of each LC was established by permutation tests. The reliability of a FC measure/mood item contributing to a corresponding LC was established by bootstrap ratio (BSR, computed by dividing each pair of saliences by its bootstrap estimated standard deviation, akin to a *z*-statistic).

Next, we computed 𝐿*_X_* and 𝐿*_Y_* by projecting 𝑋 and 𝑌 onto their respective saliences 𝑉 and 𝑈:

*𝐿_X_* = *X* × *V*

*𝐿_Y_* = *Y* × *U*

𝐿*_X_* and 𝐿*_Y_* are individual FC scores and mood scores, and reflect the participants’ individual subcortical FCs and mood symptom profile contribution to each LC (akin to factor scores in principal components analysis).

Finally, in order to infer the contribution of the original variables to the LCs’ structure, we computed Pearson’s correlations between 𝑋 and 𝐿*_X_*, as well as between 𝑌 and 𝐿*_Y_*, yielding FC loadings and mood loadings. These loadings reflect the direct contribution of a predictor to the predictor criterion independently of other predictors, which can be critical when predictors are highly correlated between each other (i.e., in presence of multicollinearity) (5). The confidence intervals on the loadings were established by a bootstrap procedure to determine the significance of the loadings.

# Supplementary results

**Identification of covariance patterns after scrubbing**

The PLS analysis was repeated in the main sample after scrubbing was applied in the MRI processing. The covariance pattern was replicable after scrubbing. Only the first LC (LC1) survived the permutation test, accounting for 41.5% of the FC-mood covariance, with significant association (*r* = 0.40, *P* = 1.20×10^-6, Figure S3A) between FC and mood scores. The subcortical FC loadings of LC1 was similar to that seen in the main findings (Figure S3B and S3C). The mood loadings of LC1 paralleled the mood loadings in the main findings (Figure S3D).

**Consideration of Other Potential Contributing Factors**

To control potential confounds (i.e. age, sex, years of education, BMI and head motion), we regressed out these confounds from both the subcortical FCs and mood symptom profile prior to the PLS analysis. We repeated PLS analysis and calculated the similarity between the obtained FC (or mood) loadings and the FC (or mood) loadings of the original PLS model. The results were highly consistent with the original PLS. The correlation between the new FC loading pattern and the original FC loading pattern was 0.96 (*P* < 0.001), and the correlation between the new mood loading profile and the original mood loading profile was 0.99 (*P* < 0.001).

# Supplementary figures

**Figure S1. Flow chart depicting the patient analysis procedure.**


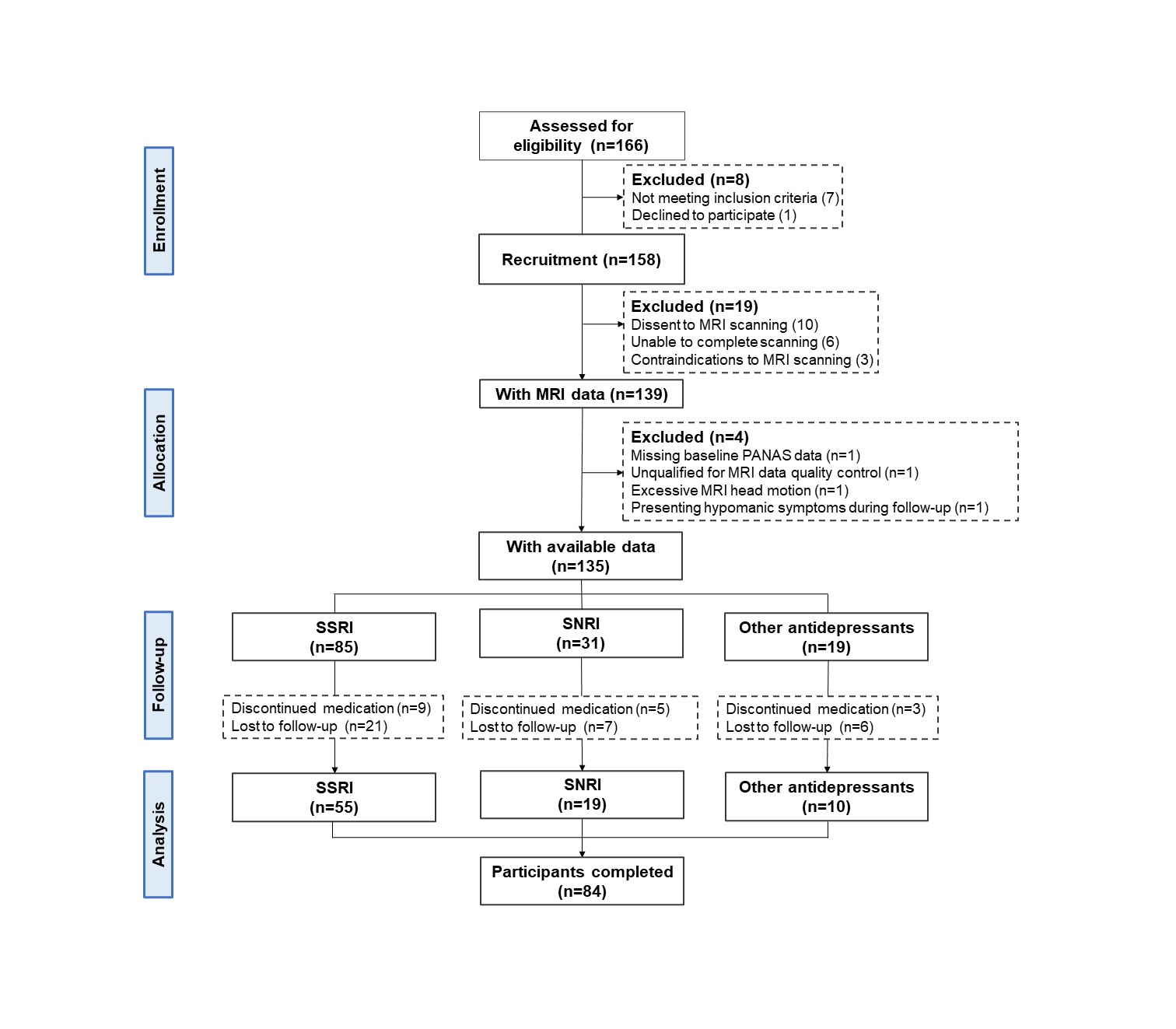


**Figure S2. Covariance explained by each latent component obtained with the PLS analysis for the TNATAD study (A) and replication sample (B).** The first component was statistically significant by permutation testing for both samples (P < 0.001).


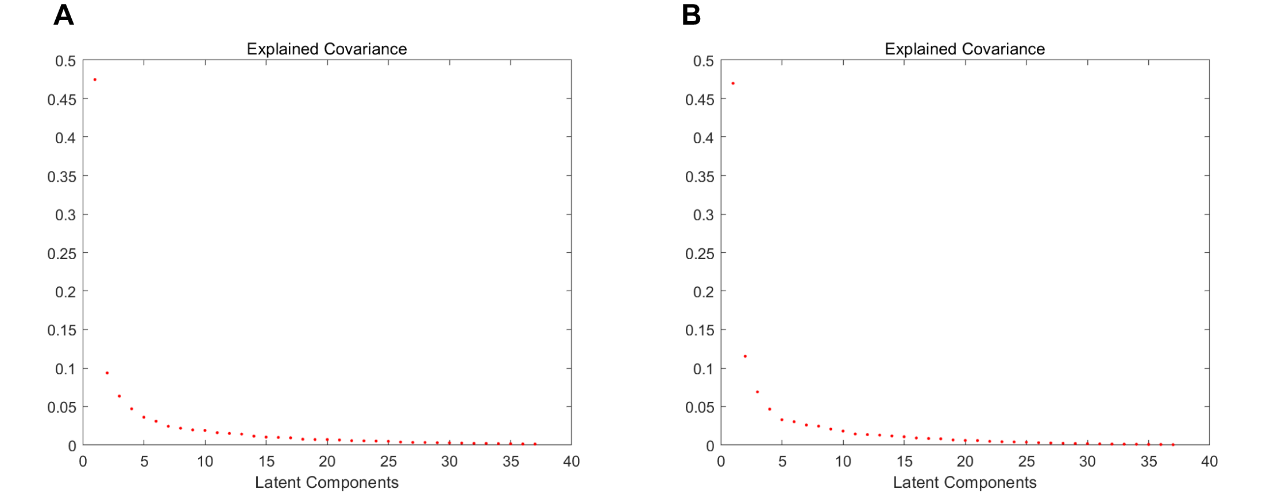


**Figure S3. FC and mood loadings in the main sample after scrubbing.**


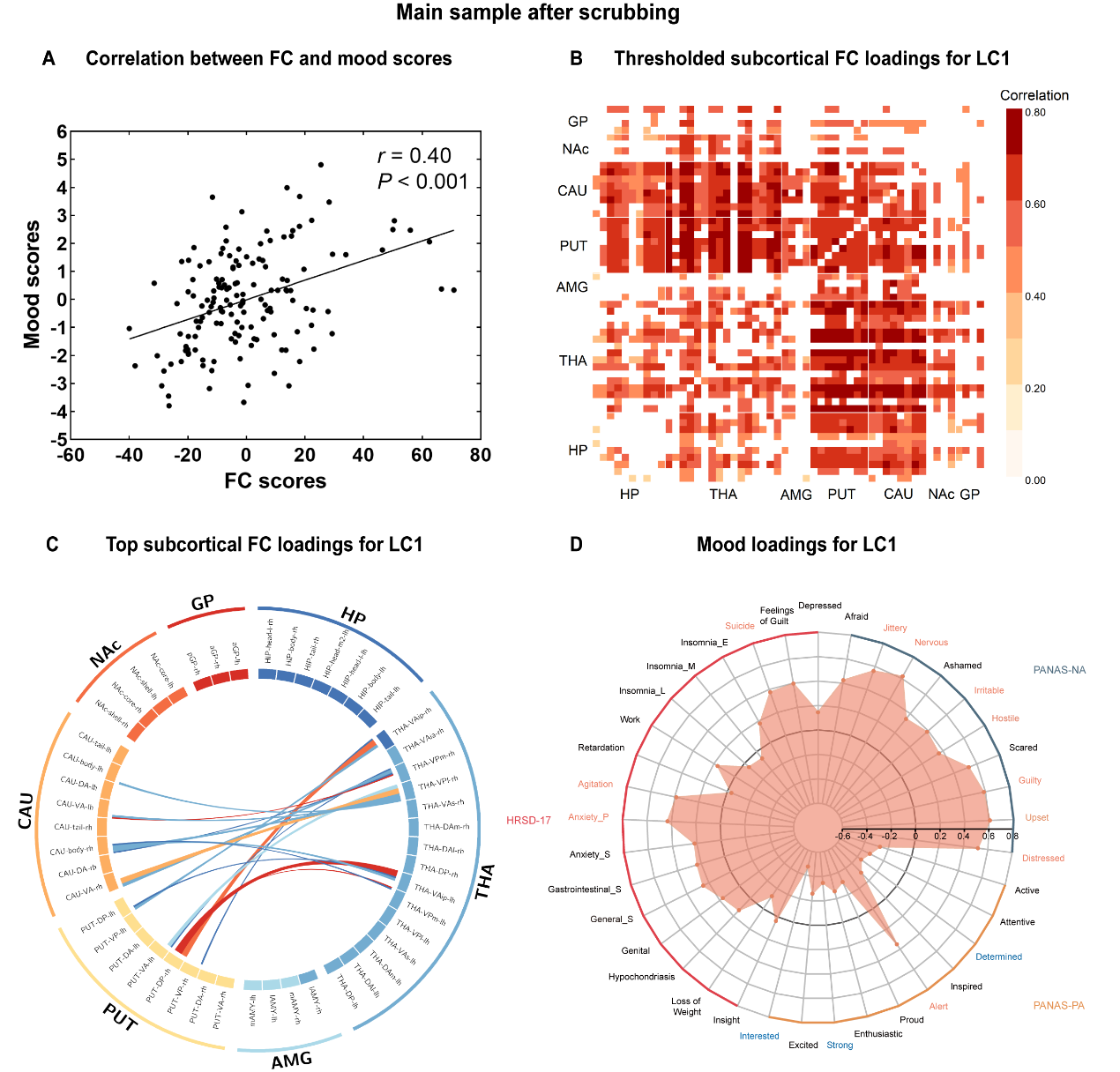


**Figure S3. FC and mood loadings in the main sample after scrubbing.** (A) Correlations between individual-specific functional connectivity (FC) scores and mood scores for the significant LC (i.e., the first LC, LC1). (B) Thresholded correlations between individual-specific original FCs and FC scores, whereby only the reliable connections (i.e., absolute value of bootstrap ratio of the FC greater than 2) that show significant FC loadings are shown. None of the thresholded correlations was negative. (C) Subcortical connections with top 5% robust FC loadings showing in the chord diagram, whereby only those connections whose absolute values of bootstrap ratio are greater than 3 and whose FC loadings are significant are shown. (D) Mood loadings for LC1. Items in PANAS-PA, PANAS-NA, and HRSD-17 are inside the orange, blue, and red arcs, respectively. Item spots inside the black circle indicates that the mood item is negatively associated with mood score and vice versa. Mood item labels with reliable contribution to the LC1 and significant mood loadings are shown in orange (or blue), indicating that higher degree of the mood item is significantly and positively (or negatively) associated with LC1 and also reliably contributed to the LC1.

Abbreviations: HP, hippocampus; THA, thalamus; AMG, amygdala; PUT, putamen; CAU, caudate nucleus; NAc, nucleus accumbens; GP, globus pallidus; pGP, posterior globus pallidus; VA, ventroanterior; DA, dorsoanterior; DP, dorsoposterior; VP, ventroposterior; THA-VAia, anterior division of inferior ventroanterior thalamus; THA-VAip, posterior division of inferior ventroanterior thalamus; THA-Vpm, medial ventroposterior thalamus; THA-Vpl, lateral ventroposterior thalamus; THA-VAs, superior ventroanterior thalamus; Insomnia_E, Insomnia Early; Insomnia_M, Insomnia Middle; Insomnia_L, Insomnia Late; Anxiety_P, Psychological Anxiety; Anxiety_S, Somatic Anxiety; Gastrointestinal_S, Somatic Symptoms (gastrointestinal); General_S, Somatic Symptoms (general).

**Figure S4. Correlations between predicted and observed FC/mood scores in the replication sample.**


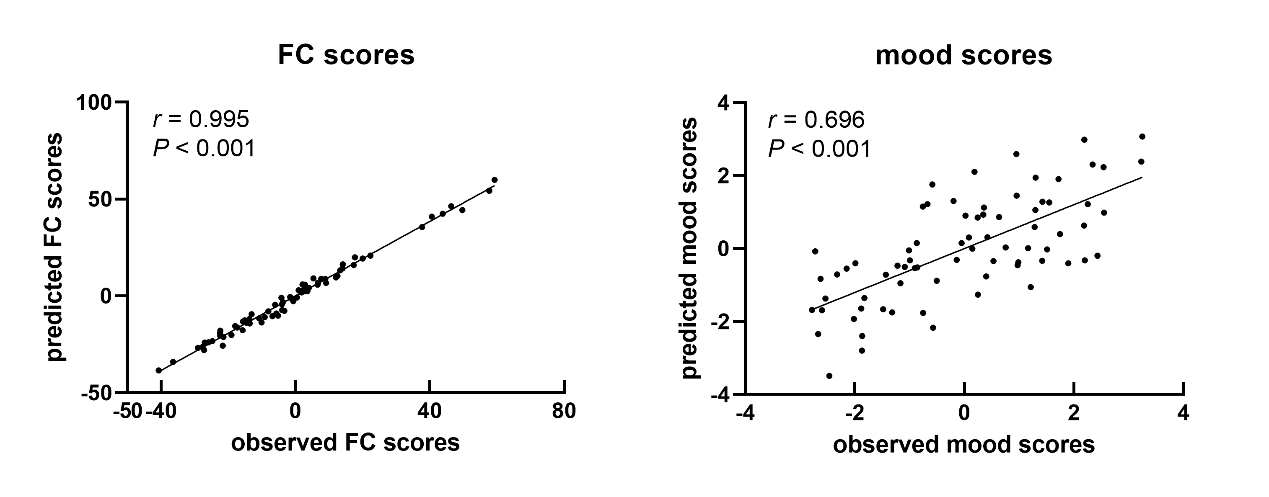


# Supplementary tables

**Table S1. Pearson correlation coefficients** **between mood score/FC score and** **demographics/clinical characteristics.** No significant correlations were found. # The number of valid values is as follows: Age of onset (n = 134), Total disease duration (n = 134), Duration of current episode (n = 103).

|  | FC score | Mood score |
| --- | --- | --- |
| age | 0.092 | -0.061 |
| BMI | 0.049 | 0.028 |
| Education years | -0.145 | 0.038 |
| Total disease duration (months) ^#^ | -0.138 | -0.053 |
| Duration of current episode (months) ^#^ | 0.041 | 0.004 |
| Age of onset^#^ | 0.138 | -0.061 |

**Table S2. Demographics and clinical characteristics and clinical measurements of depressed patients finished the 8-week of antidepressant monotherapy.**

|  | Non-remitted patients  (n = 43) | | Remitted patients  (n = 41) | | *P* value |
| --- | --- | --- | --- | --- | --- |
|  | Mean | SD | Mean | SD |  |
| Age | 30.21 | 9.36 | 30.54 | 8.14 | 0.865 |
| BMI | 21.91 | 3.20 | 22.66 | 3.80 | 0.329 |
| Years of education | 15.28 | 2.22 | 16.24 | 1.77 | 0.031 |
| Age of onset | 27.30 | 9.64 | 29.49 | 8.56 | 0.276 |
| Total disease duration (months) | 32.42 | 50.43 | 14.40 | 20.40 | 0.035 |
| Duration of current episode (months) | 4.00 | 3.08 | 3.77 | 3.19 | 0.772 |
| Total score of childhood trauma | 42.98 | 10.31 | 37.27 | 7.67 | 0.005 |
| HRSD-17 total score | 22.05 | 4.73 | 20.73 | 4.03 | 0.175 |
| PANAS_P total score | 18.12 | 4.51 | 18.10 | 4.94 | 0.986 |
| PANAS_ N total score | 27.37 | 6.81 | 26.41 | 6.38 | 0.509 |
| fluoxetine-equivalent dose (mg/day) | 35.00 | 10.12 | 30.66 | 9.60 | 0.054 |
|  | N | % | N | % |  |
| Female | 23 | 53.49 | 34 | 82.93 | 0.004 |
| First Episode MDD | 22 | 51.16 | 24 | 58.54 | 0.497 |
| Psychosis symptoms | 1 | 2.33 | 0 | 0 | 0.302 |
| Family history of psychiatric disorders | 7 | 16.28 | 6 | 14.63 | 0.835 |
| SSRI | 28 | 65.12 | 27 | 65.85 | 0.943 |

**Table S3. Prediction effect of FC score on treatment outcome.** Model 1 and model 2 were performed with general linear models. Model 3 and model 4 were performed with logistic regression. Age of onset (7), duration of current episode (7) and FC score were treated as predictors in **model 1, 3 and 4**. As Baseline HRSD-17 score was correlated with posttreatment HRSD-17 score (*r* = 0.255, *P* = 0.046), Baseline HRSD-17 score were entered in **the model 2** with the four variables above.

|  | Survived predictor | B | 95% CI | *P* | Response variable |
| --- | --- | --- | --- | --- | --- |
| Model 1 | FC score | -0.005 | -0.009, -0.001 | 0.009 | percentage reduction in HRSD-17 score |
| Model 2 | FC score | 0.133 | 0.049, 0.217 | 0.002 | posttreatment HRSD-17 score |
|  | Survived predictor | OR | 95% CI | *P* | Response variable |
| Model 3 | FC score | 0.954 | 0.922, 0.987 | 0.006 | treatment response (i.e., >50% reduction in week 8 HRSD-17 score) |
| Model 4 | FC score | 0.948 | 0.915, 0.983 | 0.004 | depression remission (i.e., posttreatment HRSD-17 score ≤7) |

# References

1. Chao-Gan Y, Yu-Feng Z. DPARSF: A MATLAB Toolbox for "Pipeline" Data Analysis of Resting-State fMRI. Front Syst Neurosci. 2010;4:13.

2. Zhang YY, Brady M, Smith S. Segmentation of brain MR images through a hidden Markov random field model and the expectation-maximization algorithm. Ieee T Med Imaging. 2001;20(1):45-57.

3. Jenkinson M, Bannister P, Brady M, Smith S. Improved optimization for the robust and accurate linear registration and motion correction of brain images. NeuroImage. 2002;17(2):825-41.

4. Yan CG, Cheung B, Kelly C, Colcombe S, Craddock RC, Di Martino A, et al. A comprehensive assessment of regional variation in the impact of head micromovements on functional connectomics. NeuroImage. 2013;76:183-201.

5. Kebets V, Holmes AJ, Orban C, Tang S, Li J, Sun N, et al. Somatosensory-Motor Dysconnectivity Spans Multiple Transdiagnostic Dimensions of Psychopathology. Biol Psychiatry. 2019;86(10):779-91.

6. Krishnan A, Williams LJ, McIntosh AR, Abdi H. Partial Least Squares (PLS) methods for neuroimaging: a tutorial and review. NeuroImage. 2011;56(2):455-75.

7. Paul R, Andlauer TFM, Czamara D, Hoehn D, Lucae S, Putz B, et al. Treatment response classes in major depressive disorder identified by model-based clustering and validated by clinical prediction models. Transl Psychiatry. 2019;9(1):187.
